# Association of sugarcane cold tolerance with stem borer resistance

**DOI:** 10.1038/s41598-026-52027-3

**Published:** 2026-05-09

**Authors:** Hannah J. Penn, James R. Todd, Keo Corak, Anna L. Hale

**Affiliations:** 1 United States Department of Agriculture, Agricultural Research Service, Sugarcane Research Unit, 5883 Usda Rd., Houma, 70360 LA USA; 2 United States Department of Agriculture, Agricultural Research Service, Genomics and Bioinformatics Research Unit, 141 Experiment Station Rd., Stoneville, 38776 MS USA

**Keywords:** cultivar resistance, fiber, freeze, Mexican rice borer, sugarcane borer, Biotechnology, Plant sciences

## Abstract

Louisiana is among the coldest sugarcane-growing locations in the world, requiring the use of cold tolerant cultivars to prevent degradation of sugar content following freezes. The most damaging pests in this system are the sugarcane borer (SCB, *Diatraea saccharalis*) and the Mexican rice borer (MRB, *Eoreuma loftini*). Both borers and cold damage are managed, in part, with resistant cultivars, with prior work indicating potential “cross-tolerance”. We reviewed the literature on commercial cultivars then used multivariate methods to assess the strength of this association, the interaction with genetics (using SSR markers), and associations with fiber, theoretically recoverable sucrose (TRS), and tonnage. SCB larval survival and weight gain were also assessed on diets from cultivars with different cold tolerance and borer resistance. Both SCB and MRB resistance were associated with good cold tolerance, but this was not mediated by fiber, TRS, or tonnage. There was no strong genetic clustering with borer resistance or cold tolerance. Further, SCB larval survival and performance did not differ among cultivar diets. These data indicate that breeding for both borer resistance and cold tolerance may be possible given the robust relationship between them. However, further evaluation of potential mechanisms and genetic linkages is required.

## Introduction

The United States of America is one of the few sugarcane-growing countries that experiences cold stress events, and as such, the use of cold tolerant cultivars is necessary to prevent degradation of sugar content following early freezes^1^. Researchers in Louisiana’s sugarcane industry prioritize cold tolerance due to regular freezes, which have compelled the industry to adjust to shorter growing and milling seasons^2,3^. Sugarcane cultivars (*Saccharum* interspecific hybrids) can achieve cold tolerance using several mechanisms such as resistance to cracking^4^, slower degradation after freezing^4,5^, tolerance of the growing point to colder temperatures^6^, ability to acclimate to cold temperatures^7^, and the ability of the plant to regrow after freezing^8,9^.

The most economically damaging insect pests of sugarcane in Louisiana are stem borers like the sugarcane borer (SCB; *Diatraea saccharalis* (Fabricius); Lepidoptera: Crambidae) and the Mexican rice borer (MRB; *Eoreuma loftini* (Dyar); Lepidoptera: Crambidae)^10^. The sugarcane borer has historically been the dominant insect pest impacting Louisiana sugarcane production and is found throughout all growing regions in the state, with an estimated economic impact of $8.0 million USD annually^11^. The MRB is a more recent pest, with the first report in Louisiana in 2008^12^. Since then, it has expanded its range at a rate of 17.69 km per year and is currently present in 23 of Louisiana’s 24 sugarcane-growing parishes (counties)^13^. The estimated cost of MRB to sugarcane production in Louisiana is $412 per hectare depending on cultivar and agronomic practices^14^. The boring damage of both species decreases the sugar content of the plant, increases fiber content making milling more difficult, and increases the chance of plant breakage and regrowth of lower-yielding bull shoots^15,16^. Further, the presence of stem borer damage is associated with increased pathogen introduction and yield losses from pathogen sucrose degradation^17^.

Like cold-tolerance, yield losses from stem borers can be managed in part by using resistant cultivars^5,18,19^. The mechanisms underlying dual protection against cold and stem borers may include traits commonly found in variety releases like fiber content^2,20,21^ and maturity date^22^. Fiber content may prevent stalks from cracking during physical compression or a freeze^4^ in addition to delaying or preventing borer entry^20,23,24^. Early maturing cane can be harvested prior to freezing events^25^. Similarly, maturation or ripening group can alter the extent and likelihood of borer damage^21,22,26^. Crop tonnage, a proxy for the combination of stalk population and size, may also play a role in both cold tolerance and borer resistance^9,27^. Greater yield, in part due to sugar content, has also been independently associated with lower cold tolerance^9^ and greater borer susceptibility^20,21^. Similar “cross-tolerance” between stressors has been documented for cold and drought in sugarcane as well as chilling and herbivory in other systems^5,28^. These data indicate that there is an overlap in cultivar traits that may be useful for breeding both cold-tolerance and stem borer resistance simultaneously.

Incidental breeding for “cross-tolerance” has already been observed in Louisiana. An evaluation of 30 years of SCB data in 16 cultivars found that the cold-tolerant cultivar HoCP 04-838 was the most SCB-resistant cultivar in the study while Ho 95–988 with poor cold tolerance was the most SCB-susceptible^21,29,30^. This may be due to USDA-ARS breeders in Houma, LA, having used wild relatives of sugarcane such as *Saccharum spontaneum* L. as a source of cold tolerance since the 1940s^31^. Currently grown commercial cultivars in Louisiana have progenitors from the germplasm enhancement (basic breeding) program^8^. Prior collaborative work between the USDA ARS Sugarcane Research Unit’s Basic Breeding and Entomology programs has indicated that there is a potential correlation between cold/freeze tolerance and SCB resistance in selected non-released cultivars^32^. Early-generation hybrids were identified with high SCB resistance along with vigorous growth, cane yield, and sucrose levels^32^. Further, these released SCB-resistant clones are parents that contribute to progeny with ratoon cold tolerance as far north as Starkville, MS, USA (33.45°N, 88.81°W) (unpublished, Hale).

This study reviews literature on released cultivars from Louisiana (“Ho” and “L” cultivars) and Florida (“CP” cultivars) breeding programs (Table 1) to evaluate the following objectives: (1) assess the overall potential of “cross-tolerance” of cold tolerance and stem borer resistance, (2) find genetic associations in released commercial cultivars using SSR markers, (3) evaluate potential mechanisms underpinning this “cross tolerance” by comparing published fiber content, sucrose content, and cane tonnage data across levels of cold tolerance and borer resistance^20,21,32^. This study also aimed to assess (4) if cross-tolerance was related to non-physical mechanisms altering stem borer development, SCB larval survival, and developmental success as determined in a laboratory diet assay of cultivars (Table 2) varying in cold tolerance and borer resistance^18,19,33^. Note, MRB survival was not evaluated as this invasive species was not present in the area of the facility conducting this work and appropriate containment was not available, preventing legal study of this organism.

**Table 1:** Cultivars used for analyses with both sugarcane borer *Diatraea saccharalis* (“SCB”) resistance and cold (“cold”) tolerance categories available in the literature.

Variety	Genetic Group	SCB	MRB	Cold	Fiber (%)	TRS (lbs/ton)	TCA (tons/acre)	References
CP 36–105	A	MR	NA	Moderate	NA			^74–77^
CP 44–101	A	S	NA	Good	NA			^76,78,79^
CP 47–193	A	S	NA	Poor	NA			^74,78,80^
CP 48–103	B2	S	NA	Moderate	11.7			^76,78,79,81,82^
CP 55 − 30	A	MS	NA	Poor	NA			^78,81,83^
CP 61 − 37	B2	S	NA	Moderate	11.01			^79,81,84–87^
CP 65–357	B1	MR	NA	Good	13.4	253.2	26.1	^85,87–90^
L 65–69^2^	B2	S	NA	Poor	NA			^81,83,91^
CP 70-321^1^	B2	R	MR	Good	12	253	29.9	^3,89,90,92,93^
CP 72–356	B1	S	NA	Moderate	NA			^89,90,92^
CP 72-370^3^	B2	MS	S	Moderate	12.5	256.6	28.8	^94,95^
CP 74–383	B1	S	NA	Moderate	12.2	238.2	30.1	^92,96^
CP 76–331	B1	S	NA	Moderate	NA			^92,96^
CP 79-318^4^	B1	MR	NA	Poor	13.1	254.7	27.2	^3,95,97,98^
LHo 83-153^1^	A	R	NA	Good	12.2	248.4	28.1	^3,98–101^
HoCP 85–845	B2	R	R	Moderate	13.8	247.5	32.2	^3,98,102,103^
LCP 85–384	B3	S	S	Moderate	11.6	254.5	35.2	^3,98,104,105^
CP 88-1762^2^	A	S	NA	Poor	NA			^36,106^
CP 89-2143	A	MS	MS	Good	10	257.4	62.7	^36,103,107,108^
HoCP 91-555^2^	B1	S	S	Poor	12.5			^3,98,109,110^
HoCP 91-552^3^	B1	MS	NA	Moderate	14.3	266.9	32	^96,97^
Ho 95-988^2^	B1	S	MS	Poor	11.3	261.7	34.2	^29,103^
HoCP 96–540	B3	S	MS	Good	11.6	256.9	33.8	^103,111^
L 97-128^2^	B3	S	NA	Poor	12.5	266	34.7	^103,112,113^
L 99-226^4^	B1	MR	MR	Poor	12	278.7	35.7	^103,114,115^
L 99-233^2^	B1	S	MS	Poor	13.3	243.2	33.4	^103,114,116^
CP 00-1101	A	MS	NA	Good	10	248.2	65.9	^36,117^
Ho 00-961^1^	B1	R	NA	Good	13.7	212.5	54.7	^118,119^
HoCP 00-950	B2	S	S	Moderate	12.5	299.4	28.7	^120,121^
L 01-283	B3	MR	MS	Good	11.6	277.2	33.3	^103,122^
L 01-299^1^	B3	R	MR	Good	12.6	264.1	36.1	^103,123^
L 03-371	B1	MS	MS	Poor	10.8	278.8	32	^103,124^
HoCP 04-838^1^	B2	R	S	Good	13	267.8	32.8	^30,103,125,126^
Ho 05-961	B1	MR	MS	Moderate	12.9	285.1	30.3	^103,127,128^
Ho 07-613^4^	B2	MR	MS	Poor	10.6	287.9	27.7	^129,130^
HoCP 09-804^3^	B2	MS	MR	Moderate	13.2	277.8	32.3	^131,132^
L 11-183^2^	B3	S	S	Poor	11.6	265.5	31.6	^131–133^
Ho 12-615^4^	B3	MR	MR	Poor	13	258.5	36.1	^18,19,133^
L 12-201^2^	B3	S	S	Poor	10.8	267.9	33.1	^18,19,133,134^
Ho 13-739^4^	B2	MR	MR	Poor	13	272	32.7	^18,133^
HoCP 14-885^2^	B2	S	S	Poor	11.8	270.9	38	^18,133^
L 14-267^3^	B3	MS	MS	Moderate	12.3	265.7	35.9	^18,19,133,135^
Ho 15–971	B1	S	NA	Moderate	11.6	262.6	40.3	^18,133,134^
HoL 15-508^3^	B3	MS	NA	Moderate	9.8	283.2	37.7	^133,136^
L 15–306	B3	S	NA	Moderate	11.1	268.1	38.6	^133,136^
NCo 310^1^	A	R	MR	Good	13.3			^76,78,81^

Mexican rice borer *Eoreuma loftini* (“MRB”) resistance category and average fiber (%) content also listed when available, otherwise “NA.” “TRS” = theoretically recoverable sucrose (lbs/ton) and “TCA” = tons of cane per acre; values obtained from SugarBase. “S” = susceptible, “MS” = moderately susceptible, “MR” = moderately resistance, and “R” = resistant. MCA1 cluster inclusion indicated by superscript numbers where 1: good cold tolerance, resistant to SCB; 2: poor cold tolerance, susceptible to SCB; 3: moderate cold tolerance, moderately susceptible to SCB; and 4: poor cold tolerance, moderately resistant to SCB.

**Table 2:** Selected cultivars tested in the laboratory for sugarcane borer *Diatraea saccharalis* (“SCB”) larval performance and the associated cold tolerance and sugarcane borer and Mexican rice borer *Eoreuma loftini* (“MRB”) resistance ratings.

Cultivar	Cold tolerance	SCB resistance	MRB resistance
HoCP 96–540	Good	Suspectable	Moderately susceptible
HoCP 04-838	Good	Resistant	Susceptible
HoCP 14–885	Poor	Susceptible	Susceptible
Ho 12–615	Poor	Moderately resistant	Moderately resistant

## Results

### Survey of released cultivars

#### Contingency table analyses

**Fig. 1:**
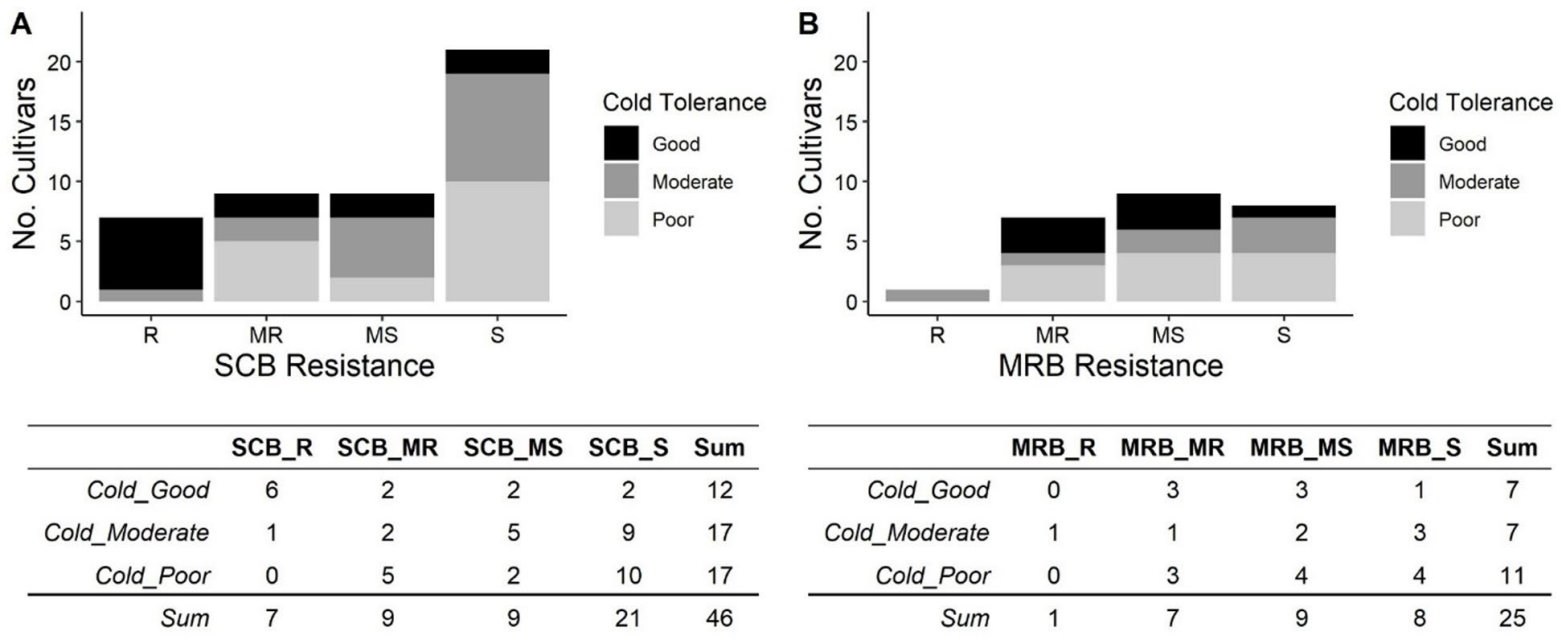
Count of cultivars for each cold tolerance category per each **(A)** sugarcane borer *Diatraea saccharalis* (“SCB”) and **(B)** Mexican rice borer *Eoreuma loftini* (“MRB”) resistance categories (“R” = resistant, “MR” = moderately resistant, “MS” = moderately susceptible, and “S” = susceptible) with associated contingency tables below each figure.

Data on both SCB resistance and cold tolerance categories was available for 46 cultivars (Table 1), with partial data collected for an additional 30 cultivars. The number of cultivars per SCB resistance categories differed significantly among cold tolerance classifications (two-tailed *P* = 0.007; Fig. 1A) using the contingency table analysis with the Fisher’s Exact Test. This was not the case for MRB resistance and cold tolerance (two-tailed *P* = 0.703; Fig. 1B), though there was only one cultivar reported to be MRB resistant.

#### Multiple correspondence analyses

**Fig. 2:**
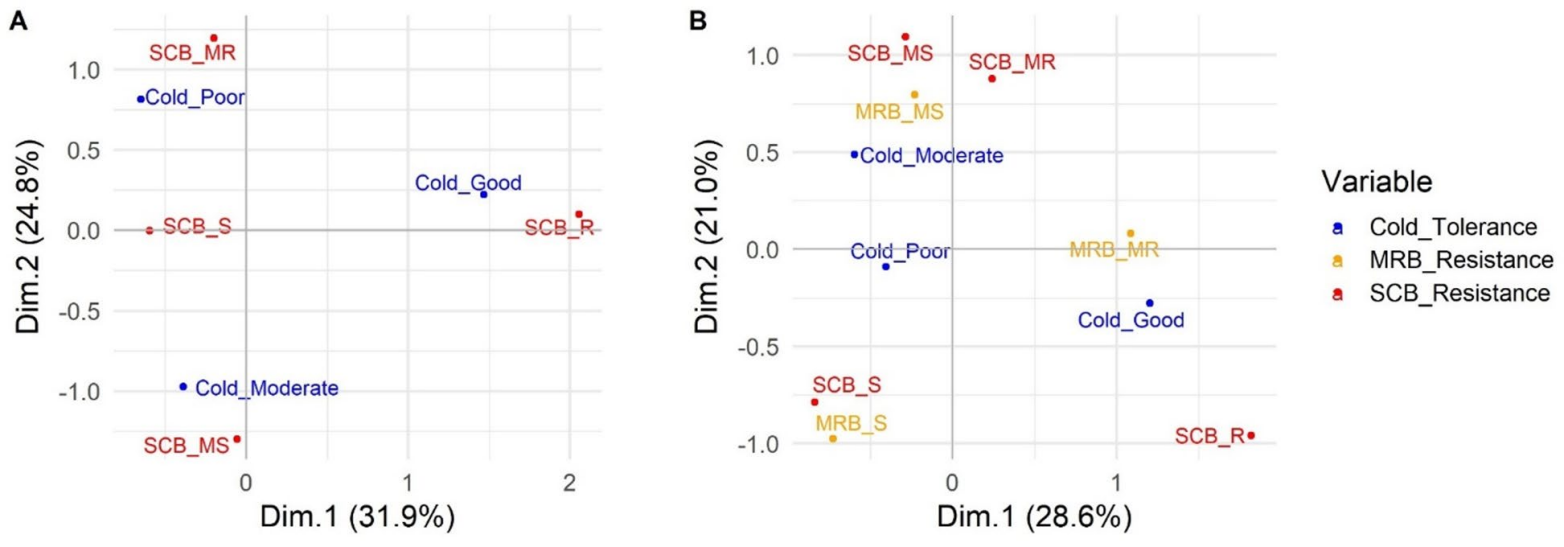
Multiple correspondence analysis of **(A)** sugarcane borer *Diatraea saccharalis* (“SCB”) resistance categories indicated in red (“R” = resistant, “MR” = moderately resistant, “MS” = moderately susceptible, and “S” = susceptible) and cold tolerance (“cold”) categories (good, moderate, or poor) in blue. **(B)** Addition of Mexican rice borer *Eoreuma loftini* (“MRB”) resistance categories in orange. Eigenvalue percentage of variance given for each dimension in parentheses.

Multiple correspondence analysis (MCA) was conducted on cultivars to further assess relationships between borer resistance and cold tolerance categories. This MCA (Fig. 2A) included all 46 cultivars with data for SCB resistance and cold tolerance. SCB resistant cultivars (Dim.1 = 2.05, Dim.2 = 0.13) were correlated with good cold tolerance (Dim.1 = 1.49, Dim.2 = 0.20), both loading into the positive section of both dimension 1 (Dim.1, 31.9% variance explained) and dimension 2 (Dim.2, 24.8% variance explained). Cultivars classified as moderately resistant, moderately susceptible, and susceptible to SCB as well as both moderate and poor cold tolerance categories loaded onto the negative sector of dimension 1 but were spread across dimension 2. However, this spread of SCB categories across dimension 2, particularly when compared to the cold tolerance categories, did not align with the degree of resistance. The second MCA (Fig. 2B) included only 24 cultivars that had information on SCB and MRB resistance as well as cold tolerance. MRB moderately resistant (Dim.1 = 1.10, Dim.2 = 0.09) cultivars were correlated with good cold tolerance (Dim.1 = 1.20, Dim.2 = -0.27), again loading into the positive section of dimension 1 (Dim.1, 28.6% variance explained).

#### Principal component analyses

**Fig. 3:**
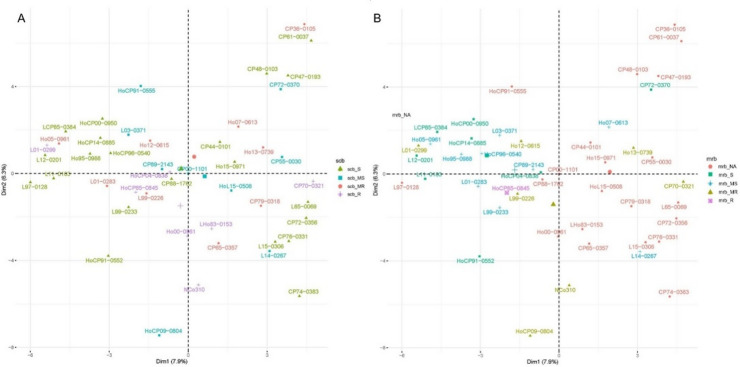
Principal components analysis (PCA) using SSR markers for individual cultivars to determine associations of **(A)** sugarcane borer *Diatraea saccharalis* (SCB) resistance categories (“R” = resistant, “MR” = moderately resistant, “MS” = moderately susceptible, and “S” = susceptible), and **(B)** Mexican rice borer *Eoreuma loftini* (MRB) resistance categories among cultivars. Eigenvalue percentage of variance given for each dimension in parentheses.

**Fig. 4:**
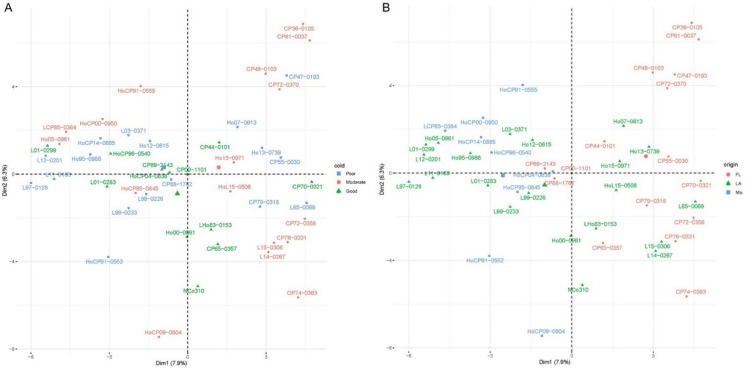
Principal components analysis (PCA) using SSR markers for individual cultivars to determine associations of **(A)** cold tolerance categories and **(B)** breeding location among cultivars. Eigenvalue percentage of variance given for each dimension in parentheses.

When the genetic groupings were assessed using principal component analysis (PCA), the first two principal components captured only 7.9% and 6.3% of the variation in the SSR molecular identity dataset (Figs. 3 and 4). Projection of individual cultivars onto these components failed to reveal any clusters enriched for SCB resistance (Fig. 3A), MRB resistance (Fig. 3B), cold resistance (Fig. 4A), or genetic subgroups (Fig. 4B). The first principal component was moderately correlated with breeding program origin (Fig. 4B). While it appears that it is also moderately correlated with MRB resistance (Fig. 4A), inspection revealed that this could be attributed to a higher proportion of missing MRB data from the Florida breeding program.

#### Associations of fiber, TRS, and TCA

**Fig. 5:**
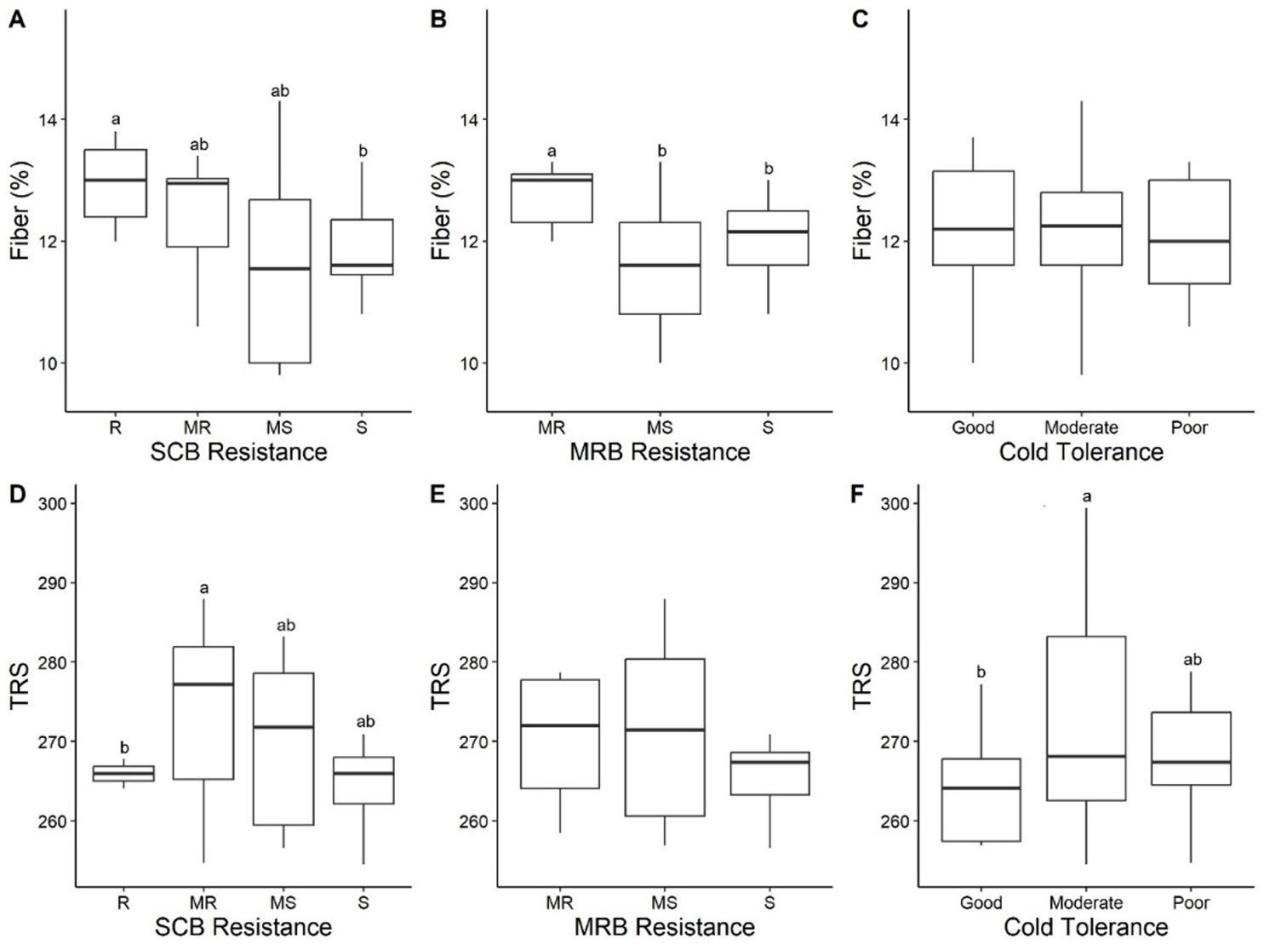
Average fiber content (%) based on each cultivar’s **(A)** sugarcane borer *Diatraea saccharalis* (SCB) resistance category, **(B)** Mexican rice borer *Eoreuma loftini* (MRB) resistance category, and **(C)** cold tolerance category. Average TRS (lbs/ton) content for each cultivar’s **(D)** sugarcane borer (SCB) resistance category, **(E)** Mexican rice borer (MRB) resistance category, and **(F)** cold tolerance category. “R” = resistant, “MR” = moderately resistant, “MS” = moderately susceptible, and “S” = susceptible. Boxplot center lines indicate the median; lower and upper box limits indicate the 25th and 75th percentiles, respectively; whiskers indicate one and a half times the inter quartile range. Different letters indicate significant differences of the means (*P* < 0.05).

Fiber content significantly differed among categories of SCB (χ^2^_(3, *N* = 38)_ = 8.190; *P* = 0.042) and MRB resistance (χ^2^_(2, *N* = 24)_ = 6.496; *P* = 0.039) but did not vary with cold tolerance (Fig. 5C; χ^2^_(2, *N* = 38)_ = 0.228; *P* = 0.892). Upon further comparison, SCB-resistant cultivars had significantly higher fiber content than SCB-susceptible cultivars (Fig. 5A), while MRB moderately resistant cultivars had higher fiber than moderately susceptible and susceptible cultivars (Fig. 5B). TRS did not differ with SCB resistance using the Kruskal-Wallis test (χ^2^_(3, *N* = 34)_ = 6.116; *P* = 0.107), but the Wilcoxon mean comparison indicated that moderately resistant cultivars had higher TRS than resistant cultivars (Fig. 5D). TRS did not differ with MRB resistance (Fig. 5E; χ^2^_(3, *N* = 23)_ = 0.036; *P* = 0.982). TRS was not associated with cold tolerance using the Kruskal-Wallis test (χ^2^_(3, *N* = 34)_ = 4.767; *P* = 0.092), but moderately tolerant cultivars had higher TRS than those with good tolerance using Wilcoxon mean comparison (Fig. 5F). TCA did not differ with SCB resistance (χ^2^_(3, *N* = 34)_ = 3.735; *P* = 0.292), MRB resistance (χ^2^_(3, *N* = 23)_ = 1.089; *P* = 0.580), or cold tolerance (χ^2^_(3, *N* = 34)_ = 0.473; *P* = 0.789).

### SCB diet incorporation of select cultivars

**Fig. 6:**
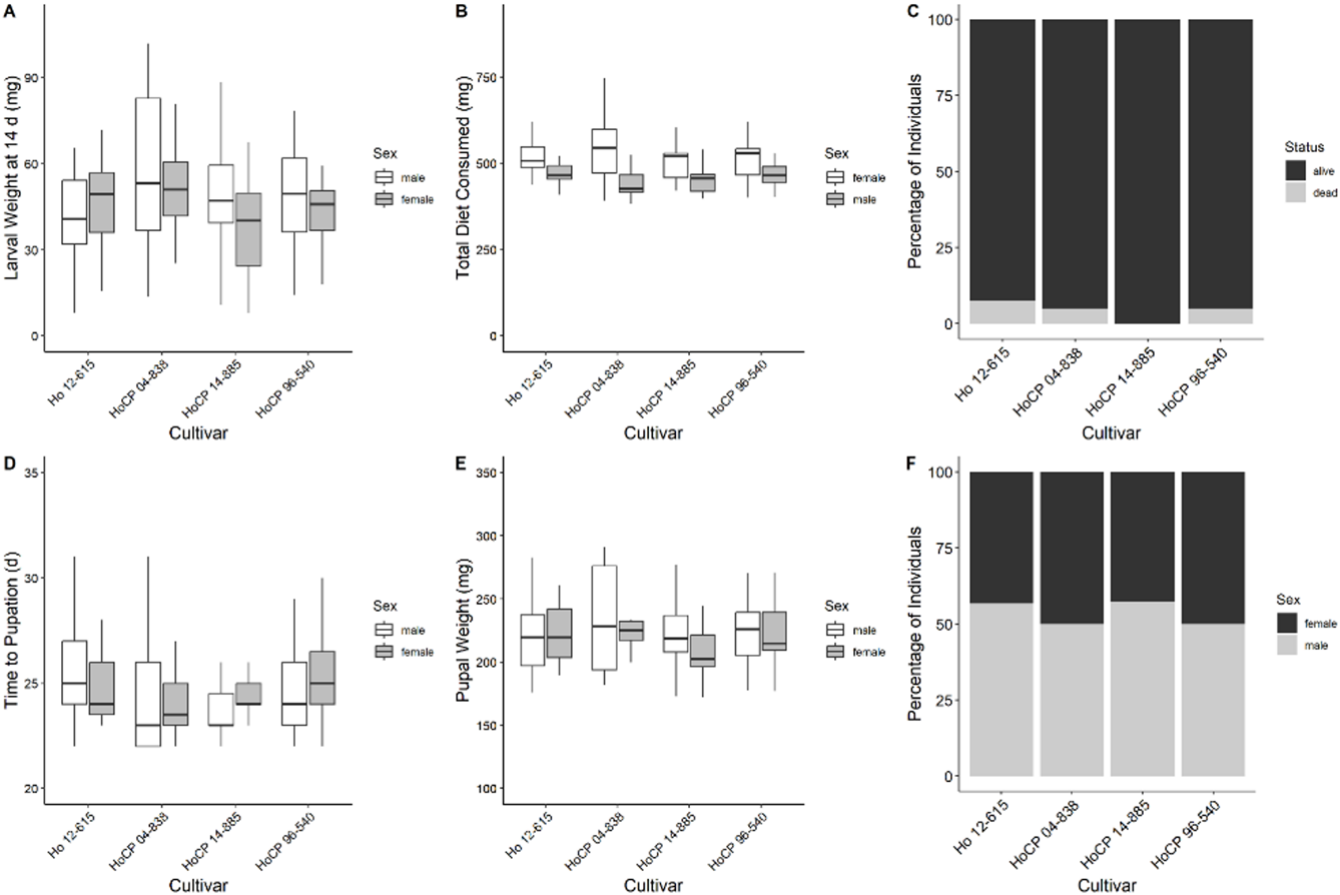
Sugarcane borer *Diatraea saccharalis* **(A)** larval weight at 14 d, **(B)** larval diet consumption, **(C)** mortality, **(D)** time to pupation, **(E)** pupal weight, and **(F)** pupal sex relative to cultivar included in the laboratory diet incorporation assay. Boxplot center lines indicate the median; lower and upper box limits indicate the 25th and 75th percentiles, respectively; whiskers indicate one and a half times the inter quartile range. Different letters indicate significant differences of the means (*P* < 0.05) for treatments.

Larval weights (Fig. 6A) did not differ with cultivar (F = 0.960; DF = 3,148; *P* = 0.416) or sex (as determined later at pupation) (F = 0.082; DF = 1,148; *P* = 0.775). Cultivar did not impact the amount of diet consumed (Fig. 6B) (F = 0.663; DF = 3,147; *P* = 0.576), but females consumed an average of 0.07 g more diet than males (F = 22.920; DF = 1,147; *P* < 0.001). Larval mortality was not altered by cultivar (Fig. 6C) (χ^2^_(3, *N* = 159)_ = 4.426; *P* = 0.219). Time to pupation (Fig. 6D) was not influenced by cultivar (F = 0.135; DF = 3,148; *P* = 0.939) but was 1.9 days longer for females (F = 4.134; DF = 1,148; *P* = 0.044). Pupal weights (Fig. 6E) were not influenced by cultivar (F = 2.032, DF = 3,148, *P* = 0.112) but were 0.04 g greater for females than males (F = 144.285; DF = 1,148; *P* < 0.001). Cultivar did not alter the sex ratio of pupae (Fig. 6F) (χ^2^_(3, *N* = 152)_ = 0.790; *P* = 0.852).

## Discussion

This study evaluated the potential for overlap of cold tolerance and stem borer resistance in commercially released sugarcane cultivars bred in two states of the United States of America. Associations of these traits with each other, genetic groupings, and a subset of potential mechanisms with the greatest availability of consistently published data were assessed based on data collected from cultivar releases and registration documents. As anticipated, there was a strong correlation between good cold tolerance and stem borer resistance for SCB and a moderate correlation with MRB resistance as well as the inverse (poor cold tolerance and stem borer susceptibility). However, correlations between moderate cold tolerance and moderate borer resistance/susceptibility were less clear. When cold tolerance and borer resistance were assessed with cultivar genetic relatedness, PCA of genetic relatedness based on SSR data only indicated a loose association with cold tolerance but not borer resistance. The relationship of cold tolerance and stem borer resistance did not appear to be mediated by fiber content, TRS, or tonnage. Additionally, SCB larval survival and performance were similar across laboratory diets made from select cultivars differing in cold tolerance and borer resistance.

There was a clear differentiation of cold tolerance using multivariate analyses. Good tolerance was separated from moderate and poor tolerance along dimension 1 while poor was separated from moderate tolerance along dimension 2 in all MCAs. Such variability in cold tolerance may be explained in part by mechanistic differences in how different genotypes handle cold stress^34^. Closely related genotypes may be responding similarly to each other because their mechanisms of cold tolerance are the same. Good cold tolerance was generally associated with good borer resistance and poor cold tolerance with borer susceptibility in the evaluated cultivars. This was particularly true for SCB, potentially as there were more observations with SCB resistance compared to MRB resistance in this dataset. However, moderate borer resistance did not cleanly group with moderate cold tolerance. The spread of the moderate categories could be due to the relative unreliability of these categories for accurately describing the traits of interest. Prior work has shown that cultivars classified with moderate borer resistance categories may change their status with time and pest pressure^21,35^. Additionally, some of the cultivars with moderate borer resistance exhibit more extreme cold tolerance ratings, altering the MCA groupings. For instance, CP 89-2143 is documented as having good cold tolerance while being moderately susceptible to borers^5,36^.

While cold tolerance and borer resistance were consistently correlated with each other, cultivars with good cold tolerance and borer resistance were not necessarily close relatives. Using PCA, Louisiana-bred cultivars were loosely associated with good cold tolerance. This makes sense as the basic breeding program in Louisiana has focused on incorporating cold tolerance for over 80 years, with some of these cultivars being released for commercial use^31^. Florida sugarcane production, being warmer year-round and with a longer growing period than in Louisiana, has not actively incorporated cold tolerance as a trait to the same extent^2,5^. Similarly, Hale et al. 2016 demonstrated that Louisiana cultivars selected for cold tolerance had less post-freeze juice degradation following a late season freeze compared to other Louisiana cultivars and to all Florida cultivars tested^2^. Further, both Louisiana and Florida programs have incorporated borer resistant parents^37^, which may help explain the lack of association of breeding location with borer resistance. But given the overall lower selection efforts for cold tolerant varieties in Florida, breeding for both cold tolerance and borer resistance should start with cultivars from Louisiana.

The potential mechanisms underlying “cross-tolerance” to cold and borers do not appear to be associated with the subset of mechanisms evaluated here – total fiber content, TRS or tonnage. TRS and tonnage were not consistently correlated with borer resistance and good cold tolerance in this study despite prior work showing higher TRS levels being negatively correlated with borer resistance and cold tolerance in select cultivars and *Saccharum spontaneum*^21,38^. As the data in this study were taken from averages, they may not account for crop age and environmental factors that also influence TRS and tonnage. Total fiber content differed with borer resistance such that greater resistance levels had higher fiber as expected^20,21^; but fiber had no association with cold tolerance, indicating that total fiber was not the mechanism of “cross-tolerance”. Sugarcane cultivars in Louisiana are not released with fiber greater than 13.8%, thus restricting the range of fiber content in many of the cultivars in this study. It’s possible that fiber levels exceeding 13.8% could be associated with cold tolerance as seen in energy canes^39^. Furthermore, total fiber does not fully capture the contributions of individual lignocellulosic components that may influence cold tolerance^40^ in addition to borer resistance^24,41,42^. However, unlike fiber, TRS, and tonnage, these data are not available for most released cultivars.

SCB larval survival and developmental success did not differ among diets made with a subset of cultivars differing in both borer resistance and cold tolerance. While this could be due to relatively low levels of sugarcane material incorporated into the diet^33,43^, this was also seen in a study comparing SCB larval success on sugarcane and energycane cultivars^36^. While cultivars differed in SCB injury, there were no differences in larval survival and weight gain among many of the canes tested. This indicates that mechanisms of borer resistance could be due to more physical traits not measured in this study such as rind hardness^20,36^ or plant traits promoting oviposition^44,45^. Similar to the lack of associations of cold tolerance with fiber and TRS, it appears that mechanisms other than those impacting larval development may be underlying borer resistance. However, additional on-plant evaluations would help elucidate these mechanisms.

Other “cross-tolerance” mechanisms not yet evaluated in tandem but common to both cold tolerance and borer resistance could include physiological differences plant structures, microbial activity, and stress responses. For instance, cultivar differences in structure-hardening fiber or silica content and location can prevent initial damage from environmental stressors and pests as well as susceptibility to future damage^20,46–48^. When damage from either freezing or borer entry does occur, these wounds become a prime colonization location by microorganisms that break down sucrose^17,49^. Such wounding from abiotic stress, borers, and accompanying pathogens can trigger plant defense pathways^50,51^. Upregulation of phenolics and plant hormones can also be associated with both borer resistance^52^ and cold tolerance^53^. Differential regulation and timing of these pathways, which have been documented as altering pathogen and environmental stress susceptibility in sugarcane^54,55^, may result in the observed correlations of cold tolerance and borer resistance^7^. As these factors may also be dependent on sugarcane cultivar^56^ and have been used to indicate sugarcane resistance to pathogens^57^, they should be investigated further to determine the potential overlapping mechanisms of extreme temperature tolerance^58,59^ and borer resistance^60^.

Aside from assessing only a sub-set of mechanisms, part of the lack of correlation between cold tolerance and borer resistance mechanisms evaluated here could be due to lack of quantitative data underlying both cold tolerance and borer resistance classifications. As the cultivars assessed here exhibited a large temporal spread in release date and personnel estimating traits (Table 1), the categorization of both cold tolerance and borer resistance might be variable. Both metrics depend on which cultivars they were compared against at time as well as the intensity of the stressor (i.e. freezing temperatures and borer populations) during the period of evaluation^9,61^. Given these limitations as well as raw data being harder to obtain for older cultivars, the categories represent subjective qualitative traits. Standardizing and consistently measuring and reporting quantitative metrics for cold tolerance as well as other cultivar traits currently reported in a descriptive fashion would benefit future assessments of breeding efforts^21^. This is currently being done for borer resistance through the consistent reported of bored internodes as a percentage of total internodes and the relative survival rate^62,63^, but these data are not available for many cultivars released prior to the 1990’s.

In conclusion, this study assessed the potential for associations between cold tolerance and stem borer (sugarcane borer and Mexican rice borer) resistance classifications for commercially released US sugarcane cultivars. Data indicated a strong association of good cold tolerance and borer resistance (especially for the sugarcane borer) as well as the inverse. However, there was little genetic relatedness of cultivars exhibiting both good cold tolerance and borer resistance. Evaluation of cultivar release data indicated that total fiber, TRS, and tonnage were not mechanisms of the “cross-tolerance” between cold tolerance and borer resistance. Even though higher fiber was seen with more borer resistant classifications, fiber did not differ among cold tolerance levels in the assessed cultivars. Further assessment found that sugarcane borer larvae performed similarly on diets of cultivars differing in cold tolerance and borer resistance. These data indicate that mechanisms unexplored by this manuscript must be underpinning the strong association of cold tolerance and stem borer resistance.

## Materials and methods

### Survey of released cultivars

To determine if sugarcane cultivars varying in cold tolerance consistently differ in their resistance to SCB, a survey of released cultivars as of 7 January 2025 was conducted. Cultivar releases, relevant literature, and reports were searched using Google Scholar for each cultivar to determine if SCB resistance and cold/freeze tolerance metrics were available. Search terms included the cultivar name and “sugarcane”, the addition of “cold or freeze tolerance”, or the addition of “borer resistance.” If both cold tolerance and SCB resistance data were not available, the cultivar was excluded from analyses. When possible, MRB resistance was recorded.

Classifications of borer resistance for either species were determined using stated classifications in the published variety releases or in studies explicitly evaluating resistance levels (Table 1). As these publications spanned from 1961 to 2026, none of the studies directly compared all cultivars. Resistance classifications within each study were relative to those cultivars evaluated over several years (and often multiple locations) of the study. Each study used a known commercial resistant and susceptible check cultivar for comparison. Many but not all studies reported the percentage of bored internodes and fewer with consistent reporting on moth production^62,63^, so we were unable to standardize based on these metrics. Given these limitations and to better match the ambiguity of the stated cold tolerance classifications, the stated resistance classification was used.

All available pedigree information for clones used in this study were downloaded in April 2025 from the USDA Sugarcanebase database, an instance of the Breedbase breeding management and analysis software^64^. Cultivar breeding program origin might be indicative of which traits were selected for given differences in location. Cultivar names beginning in “CP” indicate those that were bred in Canal Point, FL, USA; “Ho” indicates those bred in Houma, LA, USA; and “L” indicates those bred in St. Gabriel, LA, USA. As both borers and cold tolerance are stronger pressures in Louisiana compared to Florida, we expected that these traits might be associated with “Ho” and “L” cultivars. All statistical analyses were conducted in R v4.3.1^65^, and all figures were created using ggplot2^66^. The pedigree-based relationship matrix was computed with the R package AGHmatrix^67^ using the Kerr et al.^68^ method for polyploids. Ploidy for all individuals was set to 10. Hierarchical clustering of the resulting relationship matrix was performed using the hclust function in the R stats package using the default complete linkage method^65^. SSR-based molecular identity records were obtained from Sugarcanebase for 45 clones.

To evaluate the associations of stem borer resistance and cold tolerance, contingency tables were analyzed using a Fisher’s Exact test as the number of samples was low, particularly for MRB resistance. As there was only one cultivar (HoCP 85–845) that was classified as resistant to MRB in the contingency tables, this cultivar was dropped from multivariate analyses of MRB resistance. For associations between stem borer resistance and cold tolerance, multiple correspondence analysis (MCA) was conducted using the package FactoMineR^69,70^. The MCA used SCB resistance category and cold tolerance category of the cultivar. This was repeated with the addition of the MRB resistance category; though many observations had to be dropped as many cultivars were missing MRB data. Principal component analysis of the molecular identity data was conducted using the FactoMineR package v 2.11^69,70^ and plotted using factoextra v 1.0.8^71^. Resistance scores were included as qualitative supplementary variables. To determine if potential associations of borer resistance and cold tolerance were mediated by fiber content, theoretically recoverable sucrose (TRS), and cane tonnage (TCA) as an indication of plant quantity, average values for each metric per cultivar were obtained through SugarcaneBase^20,21,32^. If the data were not available there, cultivar releases and published literature were searched and all presented values averaged. Fiber, TRS, and TCA were then compared across borer resistance categories and cold tolerance categories using a Kruskal-Wallis test. Mean comparisons were conducted using the Wilcoxon test with a Benjamini & Hochberg correction for multiple comparisons.

### Borer diet incorporation of select cultivars

A diet incorporation assay was used to determine if both cold tolerance and stem borer resistance classifications impact SCB larval development and pupation success. Commercially available cultivars were selected to represent combinations of borer resistance and cold tolerance (Table 2). Leaf sheaths from the top three internodes or target internodes^24^ of each cultivar were collected from plant cane, cut into ~ 5 cm segments, and stored at -80 °C. The leaf sheaths were then lyophilized (FreeZone Freeze Dryer, Labconco, Kansas City, MO, USA) for 72 h, ground into powder with a Wiley Mill 4 (Thomas Scientific, Swedesboro, NJ, USA), and then sieved (35 mesh; 1/2 mm). The powder was stored in freezer storage bags at -20 °C until used in diet preparations and analysis of nutritional content.

When SCB borer larvae (received from Benzon on 11 July 2023) were newly hatched (15 July 2023), the sugarcane powder was mixed using with standard SCB laboratory diet (Southland Products Inc., Lake Village, AR, USA) and boiled water (600 mL water + 105 g diet + 25 g powder) for 3 min using a blender according to a combination of previously established methods and diet manufacturer instructions^18,19,72^. Approximately 10 mL of diet was dispensed into small 60 mL deli cups (2 oz) using a syringe and allowed to set at room temperature overnight (~ 12 h). Individual, newly hatched lab reared SCB larvae were placed on the diets and kept at standard rearing conditions (14:10 h Light: Dark cycle, 26 °C, 60% relative humidity) in an insect growth chamber and then evaluated for diet and larval weights after 14 days, time until pupation, pupal sex^73^, final diet weight, and pupal weight. This was replicated 40 times per cultivar.

Total diet consumed was calculated by subtracting the final diet weight from the original diet weight and minimal loss of moisture. A sub-sample of the lyophilized leaves prepared for the diet incorporation assay were sent to Brookside Laboratories, Inc. to be assessed for crude protein, fiber, and nutrient content (Table 3). Larval weights (g) at 14 d, total diet consumption (g), time to pupation (d), and pupal weights (g) were assessed using general linear models with cultivar and sex as the independent variables. Larval mortality was assessed using general models using a binomial distribution and logit link function where cultivar and sex were independent variables. The number of male and female pupae was compared among cultivars using general linear models using a binomial distribution and logit link function where cultivar was the independent variable. Model summaries were calculated using ANOVA, and mean separation calculated using Tukey HSD (α = 0.05).

**Table 3:** Results of the commercial laboratory assessment for nutrient contents of lyophilized sugarcane leaves used in the diet assay per cultivar.

Nutrient	HoCP 96–540	HoCP 04-838	HoCP 14–885	Ho 12–615
Crude protein (%)	3.9	3.57	3.46	3.44
Fiber (%)	33.52	33.84	34.71	35.51
Calcium (%)	0.395	0.337	0.26	0.278
Phosphorous (%)	0.069	0.055	0.052	0.051
Potassium (%)	0.926	1.024	1.449	1.287
Magnesium (%)	0.358	0.272	0.217	0.254
Sodium (%)	0.002	< 0.001	< 0.001	< 0.001
Iron (ppm)	91.1	91.8	82.5	137.3
Manganese (ppm)	144.9	139.6	178.2	143.8
Copper (ppm)	6.3	0.7	1.8	0.9
Zinc (ppm)	14.1	14.2	14.1	10.4

## Data Availability

The datasets used and/or analysed during the current study available from the corresponding author on reasonable request.

## References

[1] Hale, A. L. et al. Sugarcane breeding programs in the USA. *Sugar Tech.* **24**, 97–111 (2022).

[2] Hale, A. L. et al. Estimating broad sense heritability and investigating the mechanism of genetic transmission of cold tolerance using mannitol as a measure of post-freeze juice degradation in sugarcane and energycane (*Saccharum* spp). *J. Agric. Food Chem.* **64**, 1657–1663 (2016). 26885566 10.1021/acs.jafc.5b03803

[3] Eggleston, G., Legendre, B. & Tew, T. Indicators of freeze-damaged sugarcane varieties which can predict processing problems. *Food Chem.* **87**, 119–133 (2004).

[4] Irvine, J. E. & Legendre, B. L. Resistance of sugarcane varieties to deterioration following freezing. *Sugar Cane*. **2**, 1–4 (1985).

[5] Edmé, S. J. & Glaz, B. S. Field response of sugarcane genotypes to freeze stress with genotype x environment effects on quality traits. *J. Crop Improv.* **27**, 1–30 (2013).

[6] Hale, A. L., Viator, R. P., Kimbeng, C. & Veremis, J. C. Use of artificially-induced freezing temperatures to identify freeze tolerance in above-ground buds of *Saccharum* and *Erianthus* accessions. *Euphytica* **213**, 46 (2017).

[7] van Heerden, P. D. R. Differential acclimation capacity to frost in sugarcane varieties grown under field conditions. *Plant. Growth Regul.* **72**, 181–187 (2014).

[8] Hale, A. L., Viator, R. P. & Veremis, J. C. Identification of freeze tolerant *Saccharum spontaneum* accessions through a pot-based study for use in sugarcane germplasm enhancement for adaptation to temperate climates. *Biomass Bioeng.* **61**, 53–57 (2014).

[9] Ramburan, S. Optimizing sugarcane cultivar choice and time of harvest for frost-prone environments in South Africa. *Agron. J.* **106**, 2035–2042 (2014).

[10] Reagan, T. E. & Mulcahy, M. M. Interaction of cultural, biological, and varietal controls for management of stalk borers in Louisiana sugarcane. *Insects* **10**, 305 (2019). 31546775 10.3390/insects10090305 PMC6780493

[11] Wilson, B. E. Successful integrated pest management minimizes the economic impact of *Diatraea saccharalis* (Lepidoptera: Crambidae) on the Louisiana sugarcane industry. *J. Econ. Entomol.* **114**, 468–471 (2020). 10.1093/jee/toaa246 33247296

[12] Hummel, N. A. et al. Monitoring and first discovery of the Mexican rice borer *Eoreuma loftini* (Lepidoptera: Crambidae) in Louisiana. *Fla. Entomol.* **93**, 123–124 (2010).

[13] Wilson, B. E., Beuzelin, J. M. & Reagan, T. E. Population distribution and range expansion of the invasive Mexican rice borer (Lepidoptera: Crambidae) in Louisiana. *Environ. Entomol.* **46**, 175–182 (2017). 28334259 10.1093/ee/nvx036

[14] Reay-Jones, F. P. F., Wilson, L. T., Reagan, T. E., Legendre, B. L. & Way, M. O. Predicting economic losses from the continued spread of the Mexican rice borer (Lepidoptera: Crambidae). *J. Econ. Entomol.* **101**, 237–250 (2008). 18459384 10.1603/0022-0493(2008)101[237:PELFTC]2.0.CO;2

[15] Browning, H. W., Way, M. O. & Drees, B. M. *Managing the Mexican rice borer in Texas* (1989). https://oaktrust.library.tamu.edu/bitstream/handle/1969.1/160198/Bull1620a.pdf?sequence=8.

[16] de Rossato, J. A. Characterization and impact of the sugarcane borer on sugarcane yield and quality. *AJ* **105**, 643–648 (2013).

[17] Viswanathan, R. Red rot of sugarcane (*Colletotrichum falcatum* Went). *CAB Reviews*. **16**, 2563. 10.1079/PAVSNNR202116023 (2021).

[18] Salgado, L. D., Wilson, B. E., Penn, H. J., Richard, R. T. & Way, M. O. Characterization of resistance to the Mexican rice borer (Lepidoptera: Crambidae) among sugarcane cultivars. *Insects* **13**, 890 (2022). 36292838 10.3390/insects13100890 PMC9603989

[19] Salgado, L. D., Wilson, B. E., Villegas, J. M., Richard, R. T. & Penn, H. J. Resistance to the sugarcane borer (Lepidoptera: Crambidae) in Louisiana sugarcane cultivars. *Environ. Entomol.* **51**, 196–203 (2022). 34729590 10.1093/ee/nvab118

[20] White, W. H., Tew, T. L. & Richard, E. P. Association of sugarcane pith, rind hardness, and fiber with resistance to the sugarcane borer. *J. Am. Soc. Sugar Cane Technol.* **26**, 87–100 (2006).

[21] Penn, H. J. & Read, Q. D. Stem borer herbivory dependent on interactions of sugarcane variety, associated traits, and presence of prior borer damage. *Pest Manag Sci.* **80**, 1126–1136 (2024). 37855173 10.1002/ps.7843

[22] Munir, M., Afzal, M., Chattha, A. A. & Khan, H. W. A. Screening of early and medium/late maturing sugarcane varieties/lines against borer complex infestation in central Punjab, Pakistan. *J. Agric. Res.* **46**, 373–378 (2008).

[23] Tomaz, A. C. et al. Assessing resistance of sugarcane varieties to sugarcane borer *Diatraea saccharalis* Fab. (Lepidoptera: Crambidae). *Bull. Entomol. Res.* **108**, 547–555 (2018). 29198198 10.1017/S0007485317001183

[24] White, W. H. Movement and establishment of sugarcane borer (Lepidoptera: Pyralidae) larvae on resistant and susceptible sugarcane. *Fla. Entomol.* **76**, 465–473 (1993).

[25] van Heerden, P. D. R., Eggleston, G. & Donaldson, R. A. Ripening and postharvest deterioration. In *Sugarcane: Physiology, Biochemistry, and Functional Biology* 55–84 (Wiley 2013). 10.1002/9781118771280.ch4.

[26] Sturza, V. S., da Cunha, U. S., Bernardi, D., da Greco, M. G., Nava, D. E. & C. E. & Nonpreference for oviposition of sugarcane borer (Lepidoptera: Crambidae) in sugarcane seedlings is influenced by ripening group and plant age. *Environ. Entomol.* **49**, 692–698 (2020). 32318711 10.1093/ee/nvaa039

[27] Van Rensburg, J. B. J. Plant population and cultivar effects on yield losses caused by the maize stalk borer, *Busseola fusca* (Lepidoptera: Noctuidae). *S Afr. J. Plant. Soil.* **5**, 215–218 (1988).

[28] da Silva, J. A. The importance of the wild cane *saccharum spontaneum* for bioenergy genetic breeding. *Sugar Tech.* **19**, 229–240 (2017).

[29] Tew, T. et al. Registration of ‘Ho 95–988’ sugarcane. *Crop Sci.* **45**, 1660–1661 (2005).

[30] Hale, A. L. et al. HoCP 04-838 - a new sugarcane variety for Louisiana. *J. Am. Soc. Sugar Cane Technol.* **32**, 84–85 (2012).

[31] Brandes, A. F. Cold resistant sugar cane. *Cane Growers’ Q. Bull.* **7**, 175–176 (1940).

[32] White, W. H., Hale, A. L., Veremis, J. C., Tew, T. L. & Richard, E. P. Registration of two sugarcane germplasm clones with antibiosis to the sugarcane borer (Lepidoptera: Crambidae). *J. Plant. Regist*. **5**, 248–253 (2011).

[33] Meagher, R. L., Irvine, J. E., Breene, R. G., Pfannenstiel, R. S. & Gallo-Meagher, M. Resistance mechanisms of sugarcane to Mexican rice borer (Lepidoptera: Pyralidae). *J. Econ. Entomol.* **89**, 536–543 (1996).

[34] Härter, A. et al. Cold tolerance in sugarcane progenies under natural stress. *Sugar Tech.* **23**, 508–518 (2021).

[35] Reagan, T. E., Way, M. O., Beuzelin, J. M. & Akbar, W. Assessment of varietal resistance to the sugarcane borer and Mexican rice borer. In *Sugarcane research: Annual progress report* (2008). https://huaxingtaikang.com/~/media/system/2/d/0/4/2d044706f72a7f88760135e8833500a2/entomology.pdf.

[36] Sandhu, H. S. & Cherry, R. H. Sugarcane borer, *Diatraea saccharalis* (F.) (Lepidoptera: Crambidae), injury and survival in energy cane versus sugarcane. *Sugar Tech.* **20**, 558–565 (2018).

[37] White, W. H. Cluster analysis for assessing sugarcane borer resistance in sugarcane line trials. *Field Crops Res.* **33**, 159–168 (1993).

[38] Lingle, S. E., Johnson, R. M., Tew, T. L. & Viator, R. P. Changes in juice quality and sugarcane yield with recurrent selection for sucrose. *Field Crops Res.* **118**, 152–157 (2010).

[39] Baldwin, B. S., Hale, A. L., Eason, W. A. & Morrison J. I. Phenotypic evaluation of *Saccharum* spp. genotypes during the plant-cane crop for biomass production in northcentral Mississippi. *Agriculture* **14**, 1375 (2024).

[40] Ji, H. et al. The *Arabidopsis* RCC1 family protein TCF1 regulates freezing tolerance and cold acclimation through modulating lignin biosynthesis. *PLOS Gen.* **11**, e1005471 (2015). 10.1371/journal.pgen.1005471 PMC4579128 26393916

[41] Martin, S. A., Darrah, L. L. & Hibbard, B. E. Divergent selection for rind penetrometer resistance and its effects on European corn borer damage and stalk traits in corn. *Crop Sci.* **44**, 711–717 (2004).

[42] Santiago, R., Butrón, A., Revilla, P. & Malvar, R. A. Is the basal area of maize internodes involved in borer resistance? *BMC Plant. Biol.* **11**, 137 (2011). 21999882 10.1186/1471-2229-11-137 PMC3206430

[43] Hensley, S. D. & Hammond, A. M. Jr. Laboratory techniques for rearing the sugarcane borer on an artificial diet. *J. Econ. Entomol.* **61**, 1742–1743 (1968).

[44] Pimentel, G. V. et al. Oviposition preference and larval performance of sugarcane borer in eight sugarcane genotypes. *Ciênc Agrotec*. **41**, 439–446 (2017).

[45] VanWeelden, M. T., Wilson, B. E., Beuzelin, J. M., Reagan, T. E. & Way, M. O. Oviposition preference and survival of the Mexican rice borer (Lepidoptera: Crambidae) in bioenergy and conventional sugarcane and sorghum. *Environ. Entomol.* **46**, 855–863 (2017). 28595271 10.1093/ee/nvx105

[46] Vilela, M., Moraes, J. C., Alves, E., Santos-Cividanes, T. M. & Santos, F. A. Induced resistance to *Diatraea saccharalis* (Lepidoptera: Crambidae) via silicon application in sugarcane. *Rev. Colomb Entomol.* **40**, 44–48 (2014).

[47] Bocharnikova, E. & Matichenkov, V. Silicon-induced mitigation of low-temperature stress in sugarcane. In *Agro-industrial Perspectives on Sugarcane Production under Environmental Stress* (eds. Verma, K. K. et al.) 215–229 (Springer Nature, 2022). 10.1007/978-981-19-3955-6_12 (2022).

[48] Punithavalli, M., Govindaraj, P. & Balaji Rajkumar, M. Resistance mechanism of energy canes developed from *Saccharum spontaenum* and *Erianthus arundinaceus* against sugarcane borer pests. *J. Asia-Pac Entomol.* **28**, 102408 (2025).

[49] Eggleston, G. Deterioration of cane juice—sources and indicators. *Food Chem.* **78**, 95–103 (2002).

[50] Smith, J. L., De Moraes, C. M. & Mescher, M. C. Jasmonate- and salicylate-mediated plant defense responses to insect herbivores, pathogens and parasitic plants. *Pest Manag Sci.* **65**, 497–503 (2009). 19206090 10.1002/ps.1714

[51] Bartoli, C. G., Casalongué, C. A., Simontacchi, M., Marquez-Garcia, B. & Foyer, C. H. Interactions between hormone and redox signaling pathways in the control of growth and cross tolerance to stress. *Environ. Exp. Bot.* **94**, 73–88 (2013).

[52] de Mello, U. S. et al. An overview of the transcriptional responses of two tolerant and susceptible sugarcane cultivars to borer (*Diatraea saccharalis*) infestation. *Funct. Integr. Genomics*. **20**, 839–855 (2020). 33068201 10.1007/s10142-020-00755-8

[53] Naikoo, M. I. et al. Role and regulation of plants phenolics in abiotic stress tolerance: an overview. In *Plant Signaling Molecules* (eds. Khan, M. I. R., Reddy, P. S., Ferrante, A. & Khan, N. A.) 157–168 (Woodhead Publishing,2019). 10.1016/B978-0-12-816451-8.00009-5.

[54] Sundar, A. R., Viswanathan, R., Malathi, P. & Padmanaban, P. Mechanism of resistance induced by plant activators against *Colletotrichum falcatum* in sugarcane. *Arch. Phytopathol. Plant. Prot.* **39**, 259–272 (2006).

[55] Selvarajan, D. et al. Differential gene expression profiling through transcriptome approach of *Saccharum spontaneum* L. under low temperature stress reveals genes potentially involved in cold acclimation. *3 Biotech* **8**, 2563 (2018). 10.1007/s13205-018-1194-2 PMC5864577 29581927

[56] Rao, M. J., Duan, M., Yang, M., Li, M. & Wang, L. Sugarcane rind secondary metabolites and their antioxidant activities in eleven cultivated sugarcane varieties. *Sugar Tech.* **24**, 1570–1582 (2022).

[57] de Armas, R., Santiago, R., Legaz, M. E. & Vicente, C. Levels of phenolic compounds and enzyme activity can be used to screen for resistance of sugarcane to smut (*Ustilago scitaminea*). *Australas Plant. Pathol.* **36**, 32–38 (2007).

[58] Kaura, V. et al. Physiological, biochemical, and gene expression responses of sugarcane under cold, drought and salt stresses. *J. Plant. Growth Regul.* **42**, 6367–6376 (2023).

[59] Gomathi, R. et al. Induced response of sugarcane variety Co 86032 for thermotolerance. *Sugar Tech.* **15**, 17–26 (2013).

[60] das Mercês, J. K. R. et al. Methyl jasmonate modulates non-enzymatic antioxidant defenses in sugarcane under *Diatraea saccharalis* (Fabricius, 1794) infestation. *BMC Plant. Biol.* **26**, 126 (2025). 41413445 10.1186/s12870-025-07912-w PMC12831377

[61] de Assis, H. L. B., Paiva, P. E. B., Dinardo-Miranda, L. L. & Yamamoto, P. T. Estimating the relationship of sugarcane borer larvae and crop damage based on adult captures and climate variables. *Sci. Agric.* **80**, 2563 (2023).

[62] Bessin, R. T., Reagan, T. E. & Martin, F. A. A moth production index for evaluating sugarcane cultivars for resistance to the sugarcane borer (Lepidoptera: Pyralidae). *J. Econ. Entomol.* **83**, 221–225 (1990).

[63] Milligan, S. B., Balzarini, M. & White, W. H. Broad-sense heritabilities, genetic correlations, and selection indices for sugarcane borer resistance and their relation to yield loss. *Crop Sci.* **43**, 1729–1735 (2003).

[64] Morales, N. et al. Breedbase: A digital ecosystem for modern plant breeding. *G3* **12**, jkac078 (2022). 10.1093/g3journal/jkac078 PMC9258556 35385099

[65] R Core Team. *R: A Language and Environment for Statistical Computing* (R Foundation for Statistical Computing, 2025).

[66] Wickham, H. *Ggplot2: Elegant Graphics for Data Analysis* (Springer-, 2016).

[67] Amadeu, R. R., Garcia, A. A. F., Munoz, P. R. & Ferrão, L. F. V. AGHmatrix: genetic relationship matrices in R. *Bioinformatics* **39**, btad445 (2023). 37471595 10.1093/bioinformatics/btad445 PMC10371492

[68] Kerr, R. J., Li, L., Tier, B., Dutkowski, G. W. & McRae, T. A. Use of the numerator relationship matrix in genetic analysis of autopolyploid species. *Theor. Appl. Genet.* **124**, 1271–1282 (2012). 22311370 10.1007/s00122-012-1785-y

[69] Husson, F., Josse, J. & Le, S. FactoMineR: An R package for multivariate analysis. *J. Stat. Softw.* **25**, 1–18 (2008).

[70] Husson, F., Josse, J., Le, S. & Mazet, J. *Package ‘FactoMineR’* (2017).

[71] Kassambara, A. & Mundt, F. Factoextra Extract and visualize the results of multivariate data analyses (2020).

[72] Penn, H. J., Wilson, B. E., Villegas, J. M., Richard, R. T. & Johnson, R. M. Increasing nitrogen fertilizer rates lead to greater *Diatraea saccharalis* (Lepidoptera: Crambidae) injury in select Louisiana sugarcane cultivars. *J. Econ. Entomol.*10.1093/jee/toag030 (2026). 41746803 10.1093/jee/toag030

[73] Butt, B. & Cantu, E. Sex determination of lepidopterous pupae. *USDA Agric. Res. Serv. Rep.* **33**, 1–7 (1962).

[74] Long, W. H., Hensley, S. D., Stafford, T. J., Concienne, E. J. & McCormick, W. J. New method for rating sugarcane varieties for susceptibility to the sugarcane borer in Louisiana. *Sugar Bull.* **39**, 175–178 (1961).

[75] Sund, K. A. The effects of freezing temperatures on the 1963–1964 sugar cane crop, Haft Tapeh, Iran (1967). https://www.cabidigitallibrary.org/doi/full/10.5555/19690303888.

[76] Hensley, S. D. & Long, W. H. Differential yield responses of commercial sugarcane varieties to sugarcane borer damage. *J. Econ. Entomol.* **62**, 620–622 (1969).

[77] Viator, D. P. Genetic behavior of resistance in sugarcane to the sugarcane borer, Diatraea saccharalis (F.). (Louisiana State University and Agricultural & Mechanical College, 1970).

[78] Kyle, M. L. Jr The effect of varietal resistance in sugarcane on the biology of Diatraea saccharalis (F.). (Louisiana State University and Agricultural & Mechanical College, 1968).

[79] Matherne, R. J. Culture of sugarcane for sugar production in the Mississippi Delta (1977).

[80] Abbott, E. V. Problems in sugar cane disease control in Louisiana. *Proc. Int. Soc. Sugar Cane Technol.* **11**, 739–742 (1962).

[81] Coburn, G. E. Host resistance to Diatraea saccharalis (f.)-evaluation of entries in Louisiana sugarcane varietal trials, 1969-71 (Louisiana State University and Agricultural & Mechanical College, 1974).

[82] Viator, C. Associations and distribution of some yield and quality components of sugarcane in infield yield trials (Louisiana State University and Agricultural & Mechanical College, 1976). 10.31390/gradschool_disstheses.3049.

[83] Irvine, J. E. Identification of cold tolerance in *Saccharum* and related genera through refrigerated freeze screening. *Proc. Int. Soc. Sugar Cane Technol.* **16**, 147–156 (1978).

[84] Loupe, D. T., Fanguy, H., Matherne, R. J. & Giamalva, M. J. Some varietal characteristics of the more important commercial varieties. *Sugar Bull.* **52**, 21–22 (1974).

[85] Martin, F. A., Richard, C. A. & Giamalva, M. J. Effects of freeze on juice quality of several Louisiana sugarcane varieties. *Proc. Am. Soc. Sugar Cane Technol.* **4**, 30–32 (1975).

[86] Martin, F. A., Richard, C. A. & Hensley, S. D. Host resistance to *Diatraea saccharalis* (F.): Relationship of sugarcane internode hardness to larval damage. *Environ. Entomol.* **4**, 687–688 (1975).

[87] Bessin, R. T. & Reagan, T. E. Fecundity of sugarcane borer (Lepidoptera: Pyralidae), as affected by larval development on gramineous host plants. *Environ. Entomol.* **19**, 635–639 (1990).

[88] Breaux, R. D., Fanguy, H. P., Matherne, R. J. & Dunckelman, P. H. Registration of CP 65–357 Sugarcane (Reg. 35). *Crop Sci.* **14**, cropsci19740011183X001400040039x (1974).

[89] Pollet, D. K., Reagan, T. E. & Hensley, S. D. Pest management of sugarcane insects (1978).

[90] Khanzada, A. G. Screening of sugarcane cultivars against the borers infestation. *Pak J. Agric. Res.* **17**, 368–372 (2002).

[91] Anzlalone, L. Jr, Paliatseas, E. D., Giamalva, M. J. & Chilton, S. J. P. Registration of L 65–69 sugarcane (Reg. 38). *Crop Sci.* **14**, 605 (1974).

[92] Benjamin, I. I. Composition of sugar cane juice as affected by post freeze deterioration of stalks. *Proc. Am. Soc. Sugar Cane Technol.* **6**, 11–8 (1986).

[93] Legendre, B. L. Resistance of Louisiana sugarcane to deterioration from freezing temperatures. *La. Agric.* **44**, 38–38 (2001).

[94] Pfannenstiel, R. S. & Meagher, R. L. Sugarcane resistance to stalkborers (Lepidoptera: Pyralidae) in south Texas. *Fla. Entomol.* **74**, 300–305 (1991).

[95] Faw, W. F. Sugarcane planting recommendations and suggestions for Louisiana sugarcane producers (1994).

[96] Tew, T. L. et al. Registration of ‘HoCP 91–552’ Sugarcane. *J. Plant. Regist*. **5**, 181–190 (2011).

[97] Fanguy, H. P., Garrison, D. D. & Legendre, B. L. Registration of ‘CP 79–318’ sugarcane. *Crop Sci.* **29**, 1574–1575 (1989).

[98] Eggleston, G. & Legendre, B. Mannitol and oligosaccharides as new criteria for determining cold tolerance in sugarcane varieties. *Food Chem.* **80**, 451–461 (2003).

[99] Bischoff, K. P. et al. Registration of LHo 83–153 sugarcane. *Crop Sci.* **32**, 1291 (1992).

[100] Gravois, K. A., Legendre, B. L. & Bischoff, K. P. Cultivar and crop effects of sugarcane bull shoots on sugarcane yield in Louisiana. *J. Am. Soc. Sugar Cane Technol.* **22**, 42–52 (2002).

[101] Viator, R. P., Garrison, R. Jr., Tew, T. L. Jr. & D. D., Dufrene, E. O. & Sugarcane cultivar yield response to planting date. *J. Am. Soc. Sugar Cane Technol.* **25**, 78–87 (2005).

[102] Legendre, B. L. et al. Registration of ‘HoCP 85–845’ sugarcane. *Crop Sci.* **34**, 820 (1994).

[103] Legendre, B., Birkett, H. & Day, D. The mystery of lowered purities and sugar yields during the 2014–2015 harvest season 203–206 (2015).

[104] Milligan, S. B. et al. Registration of ‘LCP 85–384’ sugarcane. *Crop Sci.* **34**, 819–820 (1994).

[105] Gravois, K. A. & Bischoff, K. P. New sugarcane varieties pay big dividends. *La. Agric. Mag*. **44**, 19–23 (2001).

[106] Singh, M. P., Sandhu, H. S., Gilbert, R. A. & Sugarcane cultivar CP 88-1762. *EDS, 2015* (2015).

[107] Glaz, B. et al. Registration of `CP 89-2143’ Sugarcane. *Crop Sci.* **40**, 577–577 (2000).

[108] Singh, M. P., Sandhu, H. S. & Gilbert, R. A. Sugarcane cultivar CP 89-2143 descriptive fact sheet: SS-AGR-122/AG137, 8/2018. *EDIS, 2018* (2018).

[109] USDA ARS, Louisiana Agricultural Experiment Station, & American Sugar Cane League of the U.S.A., Inc. HoCP 91–555, a new, high yielding sugarcane variety for Louisiana. *Sugar Bull.* **77**, 4–7 (1999).

[110] Legendre, B. L. et al. Registration ofHoCP 91–555’Sugarcane. *Crop Sci.* **40**, 1506–1506 (2000).

[111] Tew, T. L. et al. Registration of ‘HoCP 96–540’ sugarcane. *Crop Sci.* **45**, 785–787 (2005).

[112] Gravois, K., Hoy, J., Bischoff, K. P., Reagan, T. E. & Kimbeng, C. L 97–128 helps sustain Louisiana’s sugarcane industry. *La. Agric. Mag*. **49**, 11–12 (2006).

[113] Gravois, K. A. et al. Registration of ‘L 97–128’ sugarcane. *J. Plant. Regist*. **2**, 24–28 (2008).

[114] Gravois, K. A., Bischoff, K. P., Reagan, G., Hoy, J. W. & Kimbeng, C. A. L 99–226 and L 99–233: two new sugarcane varieties for Louisiana’s sugar industry. *La. Agric. Mag.* **49**, 23–25 (2006).

[115] Bischoff, K. P. et al. Registration of ‘L 99–226’ sugarcane. *J. Plant. Regist*. **3**, 241–247 (2009).

[116] Gravois, K. A. et al. Registration of ‘L 99–233’ sugarcane. *J. Plant. Regist*. **3**, 248–252 (2009).

[117] Gilbert, R. A. et al. Registration of ‘CP 00-1101’ sugarcane. *J. Plant. Regist*. **2**, 95–101 (2008).

[118] Knoll, J. E., Anderson, W. & Baldwin, B. Harvest date effects on biomass yield and quality of new energycane (Saccharum hybrids) genotypes in the Southeastern USA. *Am Soc. Agron. Ab* (2010).

[119] White, W. H. et al. Registration of ‘Ho 00-961’ sugarcane. *J. Plant. Regist*. **5**, 332–338 (2011).

[120] Tew, T. et al. Notice of release of sugarcane variety HoCP 00-950. *Sugar Bull.* **85**, 21–23 (2007).

[121] Tew, T. L. et al. Registration of ‘HoCP 00-950’ sugarcane. *J. Plant. Regist*. **3**, 42–50 (2009).

[122] Gravois, K. A. et al. Registration of ‘L 01–283’ sugarcane. *J. Plant. Regist*. **4**, 183–188 (2010).

[123] Gravois, K. A. et al. Registration of ‘L 01–299’ sugarcane. *J. Plant. Regist*. **5**, 191–195 (2011).

[124] Gravois, K. A. et al. Registration of ‘L 03-371’ sugarcane. *J. Plant. Reg.* **6**, 31–36 (2012).

[125] White, W. H. Notice of release of sugarcane variety HoCP 04-838. *Sugar Bull.* **89**, 19–21 (2011).

[126] Todd, J. R. et al. Registration of ‘HoCP 04-838’ sugarcane. *J. Plant. Regist*. **12**, 324–332 (2018).

[127] Wilson, B. E. et al. A relative resistance ratio for evaluation of Mexican rice borer (Lepidoptera: Crambidae) susceptibility among sugarcane cultivars. *J. Econ. Entomol.* **108**, 1363–1370 (2015). 26470265 10.1093/jee/tov076

[128] Todd, J. R. et al. Registration of ‘Ho 05-961’ sugarcane. *J. Plant. Regist*. **16**, 341–350 (2022).

[129] White, W. H. et al. Ho 07-613 – a potential new sugarcane variety for Louisiana. *J. Am. Soc. Sugar Cane Technol.* **34**, 61 (2014).

[130] Hale, A. L. et al. Registration of ‘Ho 07-613’ sugarcane. *J. Plant. Regist*. **16**, 351–362 (2022).

[131] USDA, A. R. S. Louisiana Agricultural Experiment Station, & American Sugar Cane League of the U.S.A., Inc. Notice of release of sugarcane variety HoCP 09-804. *Sugar Bull.* **94**, 17–18 (2016).

[132] Todd, J. et al. Registration of ‘HoCP 09-804’ sugarcane. *J. Plant. Regist*. **13**, 161–169 (2019).

[133] Judice, W. Outfield variety update: a review of the 2019 outfield harvest (2020).

[134] Pontif, M. et al. Registration of ‘L 12–201’ sugarcane. *J. Plant. Regist*. **16**, 363–377 (2022).

[135] Pontif, M. et al. Registration of ‘L 14–267’ sugarcane. *J. Plant. Regist*. **17**, 343–358 (2023).

[136] LSU AgCener. 2021 Sugarcane research annual progress report (2022). https://www.lsuagcenter.com/profiles/mdaigle/articles/page1654022714179.

